# Exergaming With Integrated Head Turn Tasks Improves Compensatory Saccade Pattern in Some Patients With Chronic Peripheral Unilateral Vestibular Hypofunction

**DOI:** 10.3389/fneur.2020.00601

**Published:** 2020-06-30

**Authors:** Jaap Swanenburg, Fabienne Büchi, Dominik Straumann, Konrad P. Weber, Eling D. de Bruin

**Affiliations:** ^1^Physiotherapy and Occupational Therapy Research Center, Directorate of Research and Education, University Hospital Zurich, University of Zurich, Zurich, Switzerland; ^2^Department of Chiropractic Medicine, Integrative Spinal Research ISR, Balgrist University Hospital, Zurich, Switzerland; ^3^Department of Neurology, University Hospital Zurich, University of Zurich, Zurich, Switzerland; ^4^Department of Ophthalmology, University Hospital Zurich, University of Zurich, Zurich, Switzerland; ^5^Department Health Sciences and Technology, Institute of Human Movement Sciences and Sport, ETH Zurich, Zurich, Switzerland; ^6^Division of Physiotherapy, Department of Neurobiology, Care Sciences and Society, Karolinska Institute, Stockholm, Sweden

**Keywords:** vestibular loss, exergaming, head turns, dynamic visual acuity, saccades

## Abstract

**Background:** This study aimed to determine whether vestibular rehabilitation using active video games (Exergames), including promoted head turns and unsupported locomotion, may facilitate vestibular compensation and gait in subjects with one-sided chronic peripheral vestibular hypofunction (cPVH).

**Methods:** 12 patients with cPVH (mean age of 65 ± 12 years, 8 male) were recruited for this study. The study consisted of a four-week baseline control period T1-T2 followed by a four-week intervention period T2-T3. The intervention included exergames that required physical tasks such as steps, weight shifts or balance control to cognitive challenges, in a virtual environment to play the game. The subjects participated in a total of 176 min of exergaming in eight sessions. Because of the changing projection direction of the game to the wall, the subjects had to turn their heads constantly while playing the game. Dynamic visual acuity (DVA) was assessed. Vestibulo-Ocular reflex (VOR) gain deficit and cumulative overt saccade amplitude (COSA) were measured with the video head-impulse test. Additionally, the functional gait assessment (FGA), Extended Timed Get-Up-and-Go (ETGUG), and the Dizziness handicap inventory (DHI), were assessed.

**Results:** DVA showed no significant group level change (*p* = 0.475, z = −0.714, d = 0.295) with a small effect size and improvements in five out of 12 subjects. Ipsilesional VOR gain did not improve (*p* = 0.157, z = −1.414, d = 0.481) on group level while there was an intermediate effect size and improvements in six out of 12 subjects. COSA got significant smaller (*p* = 0.006, z = −2.746, d = 1.354) with improvements in seven out of 12 subjects. The contralesional sides did not change. The FGA for the group significantly improved with an intermediate effect size (*p* < 0.001, z = −3.08, d = 1.617) and five individuals showed clinically relevant improvements. The ETGUG group value improved significantly with a strong effect size (*p* < 0.001, z = −2.67, d = 1.030), with seven individuals contributing to this change. The DHI showed no change (*p* = 0.172, z = −1.381, d = 0.592) neither on the group nor on the individuals' level. The game scores of the subjects improved during the intervention period of the intervention for every game.

**Conclusion:** The results of this study demonstrate that exergaming with promoted head turns facilitates vestibular compensation in some subjects with cPVH. This is the first study that shows an improvement in cumulative overt saccade amplitude after exergaming in chronic vestibular subjects.

## Introduction

Vestibular disorders are either assessed through cardinal symptoms of vestibular dysfunction, through balance testing or through diagnosing of specific vestibular disorders (1). Subjects with vestibular disorders often experience symptoms like vertigo, dizziness, visual disturbance, and imbalance (2, 3). The lifetime prevalence of vertigo alone in adults aged 18–79 years was 7.4% (4). Furthermore, subjects experience anxiety and distress of depression. Many patients suffer from cognitive impairments, problems with spatial memory and attention (5–7) and sustain repeated falls (8). Having vestibular disorders, hence, relates to reduced quality of life and it negatively affects many aspects of daily living (3).

Non-pharmacological vestibular rehabilitation is thus far the only effective method for improving symptoms related to many chronical vestibular disorders (3) and two recent systematic reviews partly confirm some of this beneficial effects of exercise-based rehabilitation (9, 10). However, both reviews point out the necessity of further non-pharmacological studies into determining the optimal protocol and effect for newly developed therapies, as also reflected in clinical guidelines (11). Therefore, the further development of non-pharmacological approaches for the treatment of vestibular disorders is warranted.

Perceptual and cognitive decline in individuals with vestibular disorders can be counteracted through sustained practice of a martial art or through exposing individuals to a virtual reality environment. Both approaches relate to improved symptoms of vestibular disorders (1, 12, 13). To reduce their sensation of dizziness, patients often move their head as little as possible while moving around (14); e.g., while walking, a strategy associated with negative effects on postural balance and walking behavior on stairs (15, 16) and on community ambulation (17). Therefore, improving gait is an important goal of vestibular rehabilitation (1, 18) and this goal may be achieved by specifically including head movements into the exercise programs (19). Gaze stabilization rehabilitation may lead to some improvements in visual acuity and vestibulo-ocular reflex in these patients (20, 21), especially when applied in early disease stages (22). Next to gaze stabilization, balance training, and walking exercise for endurance, habituation exercise is one of four main exercise categories that can be used for rehabilitation (11). Habituation exercises are used to treat symptoms of dizziness produced by self-motion or that are produced by visual stimuli and are referred to as “the reduction in a behavioral response to repeated exposure to a provocative stimulus, with the goal of reducing symptoms related to the vestibular system (11).”

Neurophysiologically, individuals should in their therapy program be provided with a series of performance tasks that require using the eyes while at the same time moving the head and body (23). A proof-of-concept study from our group showed improved functional gait following training in a virtual environment that integrated visual observation and head turning movements while physically playing the Exergames. This theory-based intervention facilitated gaze stability during head movements in older adults as measured by the computerized dynamic visual acuity (DVA) test (24). The results of this study led to the hypothesis that also chronic subjects with vestibular disorders would be able to train and improve their vestibular functioning.

Video-games that require whole-body physical activity to play the game combine motor and cognitive exercises and are labeled Exergames (25). Exergames provide a motivational and entertaining way to be physically active for patients (12, 26), and recent studies demonstrated a positive effect of virtual reality-based interventions on patients with vestibular hypofunction (27–29). This form of training theoretically allows addressing all four important exercise components for vestibular subjects (11) into one exercise program: gaze stability exercises and habituation exercises (24), balance and gait exercises (30, 31), and endurance (32).

The purpose of this pilot study is to further develop the theory-based exergame intervention and describe its effects on vestibular compensation in multiple subjects with one-sided chronic peripheral vestibular hypofunction (cPVH). We hypothesized that, similar to older adults, individual patient symptoms could be influenced based on the current available evidence (11) for, and the ecological validity of, the training content.

## Methods

### Participants

For this pilot study, 12 outpatients with one-sided cPVH were recruited in the Departments of Neurology and Otorhinolaryngology at the University Hospital Zurich (USZ). A senior neurologist identified patients with one-sided cPVH at baseline. A horizontal video head-impulse test (video HIT) to both sides was used to identify cPVH at T1. A video HIT gain (eye velocity divided by head velocity at peak head acceleration) below 0.7 was considered pathological. Exclusion criteria were; (1) walking disability (independent walking <10 meters), (2) acute pain, (3) medication reducing postural balance (4) weakness resulting from neurological problems, (5), uncontrolled cardiovascular disease (e.g.,: uncontrolled blood pressure) (6) gait disorders putatively attributed to other than primarily vestibular causes, (7) uncorrected severe visual impairment. Additionally, patients with Menière's disease or with benign paroxysmal positional vertigo were excluded. All measurements and exergame sessions took place at the University Hospital Zurich. The measurement assistants were blinded. All subjects provided written informed consent before partaking in the therapy sessions and the study was approved by the ethics committee of the Canton of Zurich under BASEC 2018-00337. Trial registration was done at ClinicalTrials.gov Identifier; NCT03536533.

### Design

Subjects were included and primarily assessed using an A-B study design (33). Vestibular function was tested on three separate test sessions T1, T2, and T3 at intervals of one month. The first month served as control period (A) between T1 and T2 whereas, the second month functioned as intervention period (B) between T2 and T3. DVA and VOR gain were used to test for chronicity (T1-T2-T3) of the clinical presentation of symptoms and functional impairment in enrolled subjects with cPVH.

### Intervention

Purpose-developed games were used in the study that are targeting to improve cognitive functions such as divided attention, working memory, inhibition, and attention shifting. Physical functions, such as balance and gait, are trained because the games are played through whole body movements in standing position and, preferably, without support from a handrail. By changing the orientation of the projection during the game-play (see Video Exergame) participants need to turn their head to successfully play the games. Additionally, in every session one game had to be played while the participant stood rotated 90° to the left or right and with head turned toward the projection. The detailed exercise protocol is described in detail elsewhere (24). During the intervention, the subjects received one-on-one supervision from the research assistant. In total, eight exergaming sessions were accomplished for each patient over the 4-week intervention period T2–T3. The training principles of progression and overload were applied (31). Each session lasted for 40 min, with an actual exercise duration of 22 min. The total cumulative intervention exercise time of 176 min slightly exceeded the recommended time (150 min) for an efficient virtual reality intervention (12, 24). The certified medical device exercise system Senso (dividat, Schindellegi, Switzerland), a pressure-sensitive plate, was used for the training (24). The Senso pad provided the participants with real-time visual and auditory feedback. The visual angle of the projected game was ±81° in width and ±54°in height. To promote head movements during the exercises the projector was either rotated ±45 degrees horizontally or ±15 degrees vertically. A remote-controlled power panner (Maxwell MPR−202) was used to move the projector with 6° per second horizontally and 2° per second vertically. Misery Score (MISC) was used to determine whether the training could be continued in a given level. The MISC is a simple scale to gather nausea symptoms. It is categorized in 1 = no nausea, 2 = initial symptoms, but no nausea, 3 = mild nausea, 4 = moderate nausea, 5 = severe nausea, and 6 = vomiting. In the present study, MISC 1-3 was accepted during training. A MISC score 4 and more would have led to a training stop (27) or to lowering the exercise level of difficulty. The exercise set-up is shown in a Video 1 (see Video Exergame).

### Games

Four different games were used during the intervention session. As a warm-up, the game “Simple” was used. This game trains focused attention. Thereafter the game “targets” was played. This game trains goal-oriented reacting. Cognitive flexibility was trained with the third game “flexi.” The final game was the game “snake,” which focuses on visual processing. The difficulty of all games was progressively adapted by speeding up the game according to the performance of the player, or in the fourth game “snake” the length of the snake increased progressively (24) screen images of all games in the Supplementary File 1.

### Measurements

#### DVA

DVA measures visual acuity during head movement relative to baseline static visual acuity (SVA) (34, 35). Passive head impulses were performed using a DVA testing system that consisted of a personal computer with a 19-inch monitor (1280 × 1024 pixels, 75 Hz) and a Sparkfun velocity sensor (Sparkfun Electronics, Boulder, Colorado), which was fixed on a headset to the participant‘s head (36, 37). The test gives information on the vestibulo-ocular reflex (VOR) performance, mainly of the semicircular canal function (36) and has change in visual acuity, when moving the head compared to static acuity, as output (38). For horizontal testing, the head rotation was assessed in both left and right directions, for each patient. (39). One of two experienced examiners conducted testing.

#### VOR Gain

The video head-impulse-test measurements (vHIT) were used to assess the VOR gain (40, 41). This test gives information about the function of the six semicircular canals individually by measuring the eye rotation response to an abrupt head rotation in the plane of the canal (41). The measurement protocol is described in detail in previous studies (41, 42). An ICS Impulse, Natus Medical Inc., Taastrup, Denmark was used to measure the horizontal canals. One of two experienced examiners conducted testing.

#### Cumulative Overt Saccades Amplitudes (COSA)

The characteristic saccade pattern responses are highly effective in distinguishing patients with unilateral vestibular loss compared to controls (43). OtosuiteV 2.0 (GN Otometrics) was used to re-analyse VOR gains. To qualify corrective saccades we used a custom-written MATLAB (The MathWorks, Natick, MA, USA) routine. This provided cumulative overt saccade amplitudes (44–46). Saccades that occurred after the head-impulse are defined as “overt” (46).

#### Functional Gait Assessment (FGA)

FGA is used to measure disturbances in balance and gait (47) and includes the following 10 items (48): (1) Gait on level surface, (2) Change in gait speed, (3) Gait with horizontal head turns, (4) Gait with vertical head turns, (5) Gait and pivot turn, (6) Step over obstacle, (7) Gait with narrow base of support, (8) Gait with eyes closed, (9) Ambulating backwards, (10) Steps. Each item was scored on a 4-point ordinal scale with scores of 0, 1, 2, and 3 (Maximum total score = 30). Higher scores represented better balance and gait capacity (47). The minimal detectable change is 6 points for the FGA (49) and was used to assess change on individual level.

#### Extended Timed Get-Up-And-Go (ETGUG)

The ETGUG test (50) measures times in seconds to complete a series of functionally important tasks and the overall time needed (seconds) to complete the test with a multimemory stopwatch. ETGUG test included the following tasks: (1) Rising from a chair, (2) Initiating gait, (3) Walking 6 meters straight, (4) Turning around, (5) Walking back, (6) Sitting down again. The time measured during each task and the overall time depicted the functional mobility of the participant (51). With an ICC of 0.97 for the test-retest reliability the test exhibits excellent reliability in vestibular patients (52). For the minimal detectable change we used the 4 seconds difference between patients with both-sided vestibular loss and healthy controls reported in literature (52).

#### Dizziness Handicap Inventory (DHI)

The DHI German version is a validated, 25-item-self-report questionnaire to evaluate the self-perceived handicapping effects caused by dizziness (53). The DHI is categorized in three domains; functional (9 questions, 36 points), emotional (9 questions, 36 points), and physical (7 questions, 28 points). Subjects answer the questions with no (0 points), sometimes (2 points), and yes (4 points) (54). The higher the score, the greater the handicap caused by dizziness. The cut-off value of clinical relevance is 9 points (53).

#### Simulator Sickness Questionnaire (SSQ)

The SSQ questionnaire was used to assess virtual reality users' level of sickness symptoms (55). It contains 16 items which had to be weighed by the participants in terms of severity on a 4-level scale with the options including “none,” “slight,” “moderate,” and “severe.”(56). (Minimum score = 0, Maximum total score = 48). After each exercise session and a few hours later the same day, the subjects completed the SSQ.

#### Game Scores

Game scores and therapy attendance of the exercise sessions were recorded to monitor progression and adherence to the training plan.

### Statistical Analysis

Descriptive statistics were used to describe the community dweller's characteristics. Normality of data distribution was tested using the Shapiro-Wilk test. In case of non-normal distribution, the Wilcoxon test was chosen to test the differences between measurement time points. Additionally, for the effect size, Cohen's d (d) values were calculated. Cohen (1988) reports the following intervals for d: 0.1 to 0.3: small effect; 0.3 to 0.5: intermediate effect; 0.5 and higher: strong effect. For sub analysis of FGA and ETGUG tasks a Bonferroni correction was used. Game scores and SSQ were described with descriptive statistics. Additionally a scatterplot with VOR gain and COSA before and after intervention with associated linear regression line was plotted.

IBM SPSS Statistics 25 for Windows (Inc; Chicago, Illinois) was used for all the statistical analyses.

## Results

The scores of 12 (mean age of 64.9 ± 11.7 years, 8 male) subjects with one-sided cPVH were analyzed. The mean weight of the subjects was 69.6 ± 10.3 kg and the mean height 168.0 ± 7.7 cm. Two DVA measurements at T1 could not be executed due to technical failure of the measurement device (PT 7 and PT 12). Four subjects received physiotherapy whereof one patient received cervical physiotherapy before the intervention program (PT 3) and the other three subjects were treated for lower extremity issues. Assessed by the MISC no exercise session had to be stopped. There were no adverse events.

### Control Period T1–T2

During the control period T1–T2 the DVA lesion side (*p* = 0.646, z = −0.459, d = 0.188) and the contralateral side (*p* = 0.944, z = −0.070, d = 0.029) were stable and showed no significant change. A similar result was found with the vHit gain, assessed with the head impulse paradigm, of the affected (*p* = 0.694, z = −0.393, d = 0.161) and the contralateral side (*p* = 0.695, z = −0.393, d = 0.161) both showing no significant change.

### Intervention Period T2–T3

#### Vestibular Compensation

**Figure 1:**
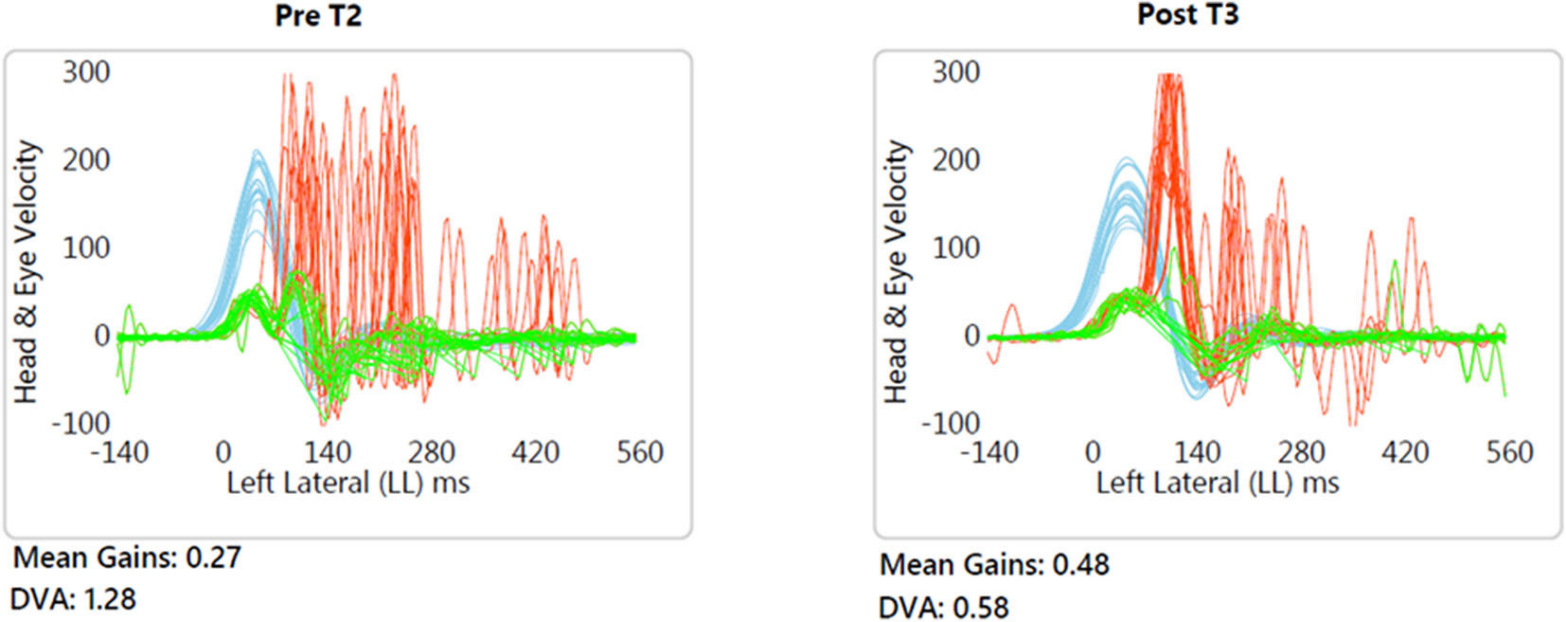
Typical example (patient no. 9) of improved VOR gain and reduced compensatory saccade variability. Blue, head velocity; Green, eye velocity; Red, compensatory saccades.

**Figure 2:**
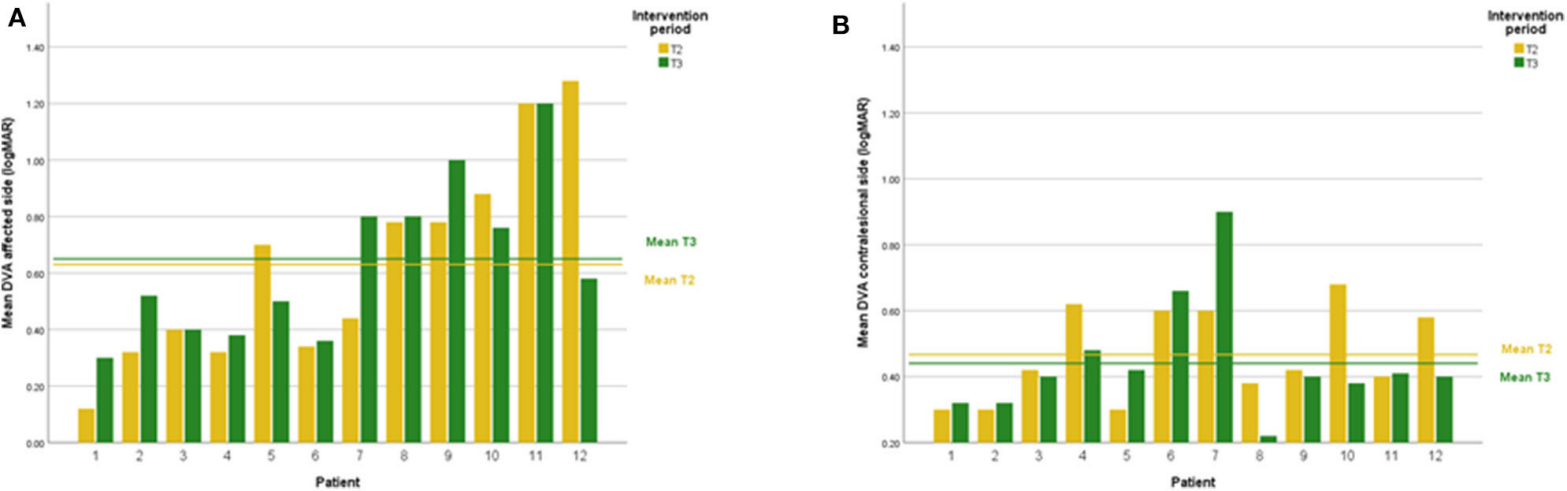
**(A)** Individual DVA results lesion side at T2 and T3. **(B)** Individual DVA results contralesional side at T2 and T3.

**Figure 3:**
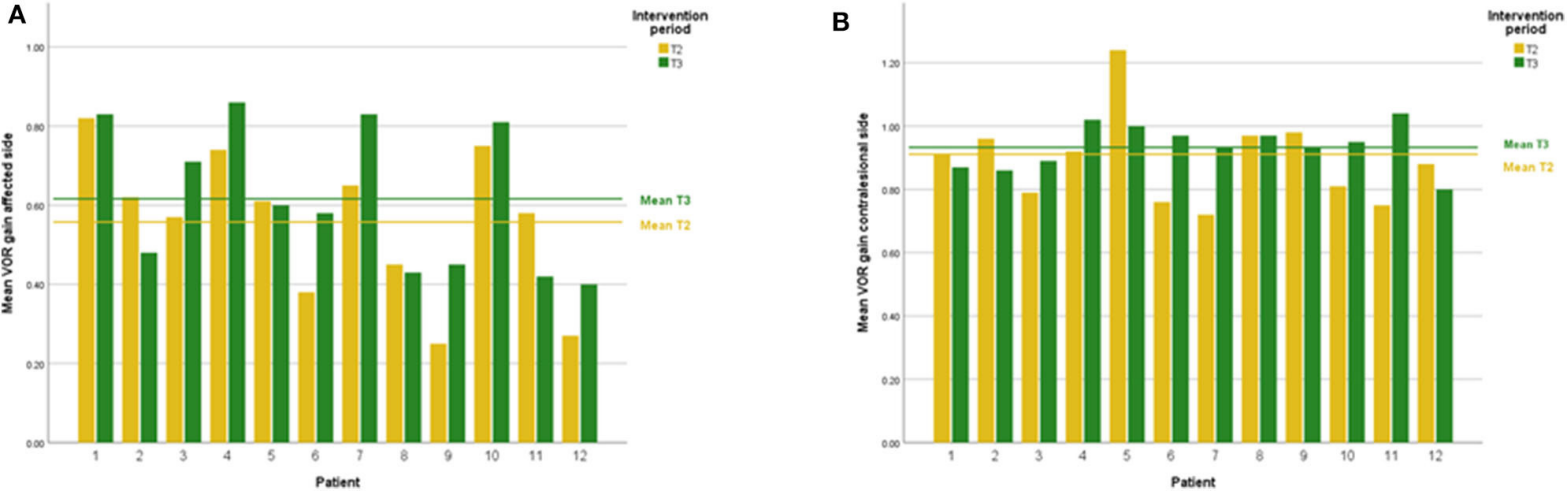
**(A)** Individual VOR gain results lesion side at T2 and T3. **(B)** Individual VOR gain results contralesional side at T2 and T3.

**Figure 4:**
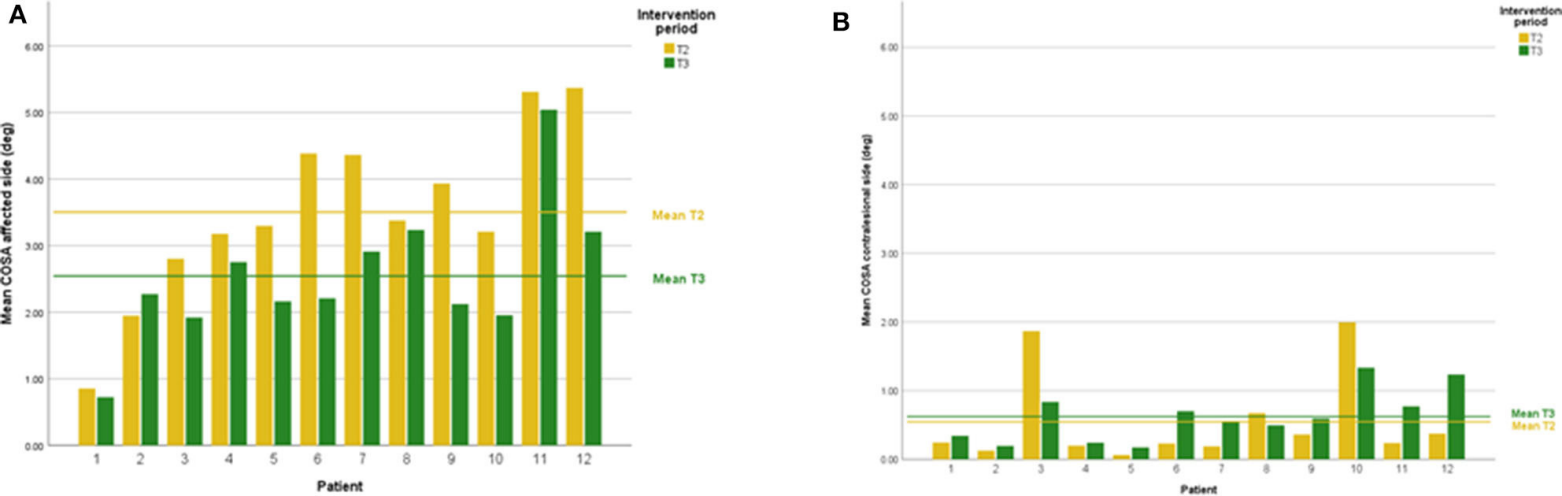
**(A)** Individual COSA results lesion side at T2 and T3. **(B)** Individual COSA results contralesional side at T2 and T3.

DVA showed no significant change (*p* = 0.475, z = −0.714, d = 0.295) with a small effect size and improvements in five out of 12 subjects. In addition, VOR gain at the ipsilesional side did not improve (*p* = 0.157, z = −1.414, d = 0.481) on group level and there was an intermediate effect size and improvements in six out of 12 subjects. COSA got significant smaller (*p* = 0.006, z = −2.746, d = 1.354) with improvements in seven out of 12 subjects. The contralesional sides did not change. A graphical plot example of an impulse test can be seen in Figure 1. All data of vestibular compensation can be found in Table 1. Results and changes of each individual participant can be found in Table 2 and Figures 2–4.

**Table 1:** Results of the Vestibular compensation pre and post intervention (T2–T3).

	**DVA (logMAR)**				**VOR Gain**				**COSA**			
	**Lesion side**		**Contralateral side**		**Lesion side**		**Contralateral side**		**Lesion side**		**Contralateral side**	
	**T2**	**T3**	**T2**	**T3**	**T2**	**T3**	**T2**	**T3**	**T2**	**T3**	**T2**	**T3**
Mean	0.630	0.633	0.470	0.439	0.518	0.667	0.911	0.933	3.502	2.543	0.546	0.623
SD	0.367	0.2.80	0.147	0.140	0.213	0.162	0.148	0.078	1.301	1.040	0.666	0.380
Z	−0.714		−0.512		−1.414		−1.070		−2.746		−0.941	
*p*	0.475		0.609		0.157		0.347		0.006^*^		0.941	

*SD, standard deviation; Range, min/max; Z, p, Wilcoxon Signed Rank Test*;

* *p < 0.05 significant*.

**Table 2:** Individual descriptive data.

**Nr**	**Age range**	**Diagnosis**	**Lesion side**	**Medication**	Additional non-vestibular therapy^*^	**DVA**	**VOR gain**	**COSA**	**FGA**	**ETGUG**	**DHI**
						**Change ± 0.05**	**Change ± 0.05**	**Change ± 0.1^**°**^**	**Change ± 6 points**	**Change ± 4 s**	**Change ± 9 points**
1	55-60	cPVH	Right	None	None	+	~	+	~	+	~
2	60-65	cPVH	Left	Aspirin Cardio	None	+	–	~	~	~	~
3	50-55	cPVH	Right	None	Physiotherapy cervical	~	+	–	~	+	+
4	45-50	cPVH	Right	Insulin	None	+	+	~	+	+	~
5	55-60	cPVH	Left	Cholesterol-lowering	Physiotherapy lower extremity	–	~	+	~	+	~
6	60-65	cPVH	Right	Aspirin Cardio, Antihypertensive	None	~	+	+	~	~	~
7	80-85	cPVH	Right	Aspirin Cardio Antihypertensive, Cholesterol-lowering, rare: temesta	Physiotherapy lower extremity	+	+	+	+	~	~
8	65-70	cPVH	Left	Aspirin Cardio, Cholesterol-lowering	None	~	~	–	~	~	+
9	80-85	chronic dizziness under stress	Left	Antihypertensive drug, Aspirin Cardio	None	+	+	+	+	+	~
10	75-80	cPVH	Left	Antihypertensive drug	Physiotherapy lower extremity	–	~	–	+	+	~
11	60-65	cPVH	Left	Aspirin Cardio	None	~	–	+	+	+	+
12	55-60	chronic neuritis vestibularis	Left	None	None	–	+	+	~	~	+

*+, improvement, –, deterioration, ~, no change by minimal detectable values or by clinical judgment in case of VOR gain*.

* *Additional non-vestibular complaints related therapy received during this study*.

**Figure 5:**
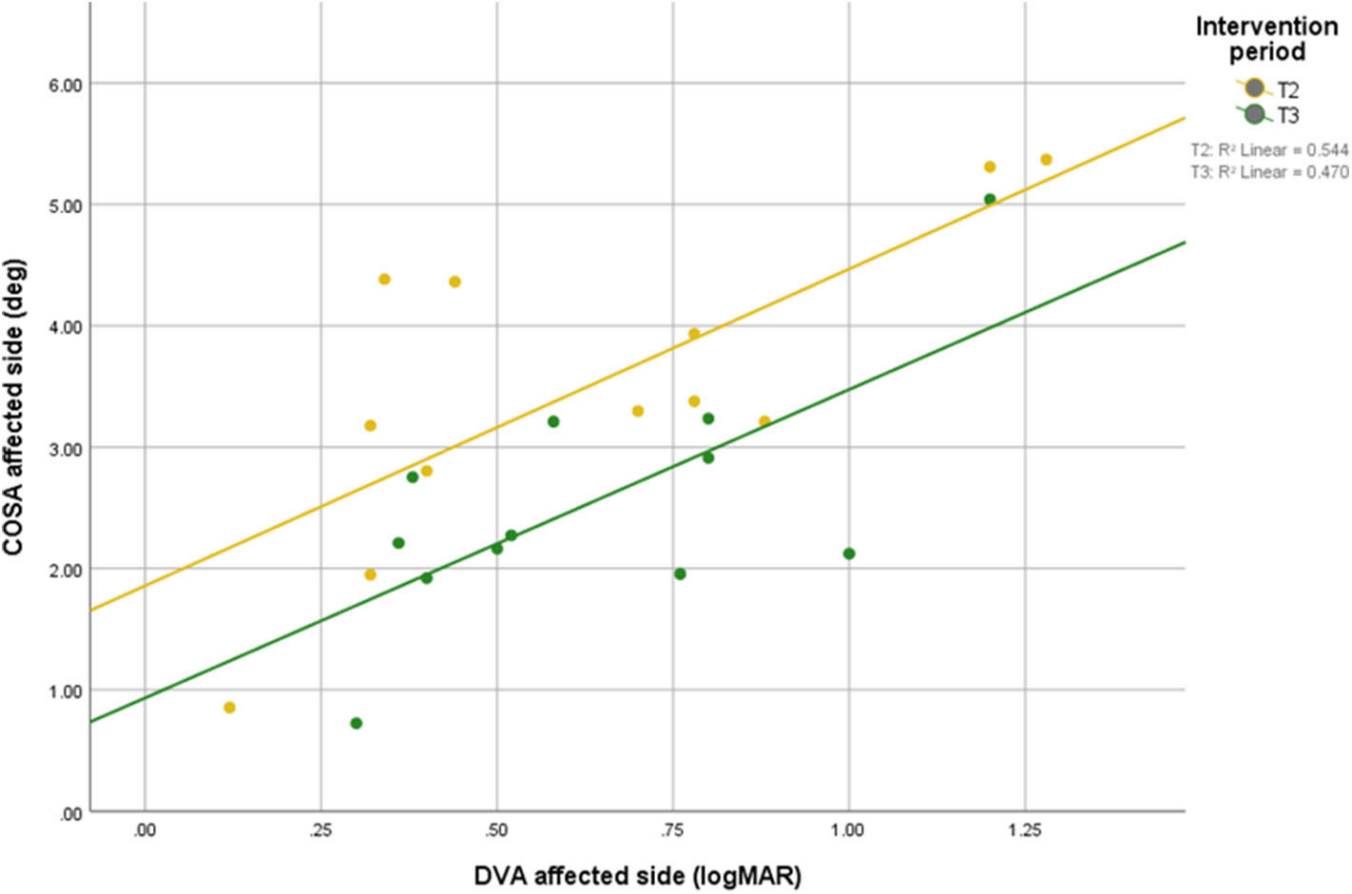
Relationship between DVA and overt saccade amplitude before and after intervention with associated linear regression line.

**Figure 6:**
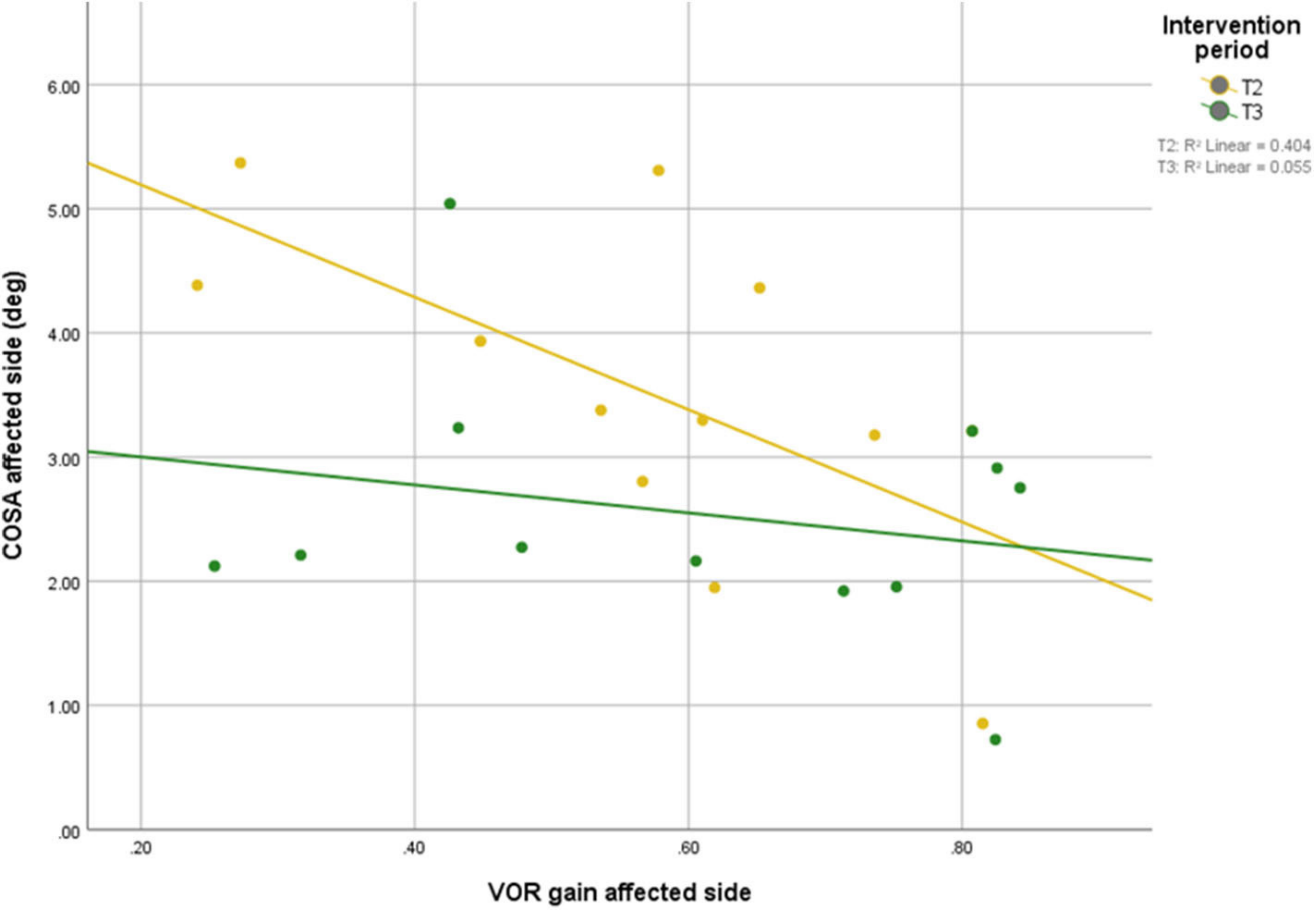
Relationship between VOR gain and overt saccade amplitude before and after intervention with associated linear regression line.

Scatterplots between DVA and COSA before and after intervention with associated linear regression lines are shown in Figure 5. A scatterplot with VOR gain and COSA before and after intervention with associated linear regression line is shown in Figure 6.

#### Gait Function

The FGA showed significant group-level improvement with a large concomitant Effect Size (*p* < 0.001, z = −3.08, d = 1.617). The total points improved from 19.58 at T2 to 26.17 points at T3. Five subjects who improved more than the minimal detectable change of six points drove this change. For the sub-tasks gait with horizontal head turns (*p* < 0.001), gait with eyes closed (*p* < 0.001), and ambulating backward (*p* < 0.001) significantly improved. All results of all FGA tasks can be found in Supplementary Table 1.

The ETGUG improved significantly on the group level with a strong effect size (*p* < 0.001, z = −2.67, d = 1.030). The total time decreased from 19.95 s at T2 to 15.61 s at T3. Seven subjects improved more than 4 seconds. The ETGUG sixth task “slow down, stop, turnaround, and sit down” significantly (*p* < 0.001) improved for the group of twelve individuals. All results of all individual ETGUG component tasks can be found in the Supplementary Table 2.

#### Dizziness

There was a non-significant decrease in DHI after the intervention session albeit there was a strong effect size (*p* = 0.172, z = −1.381, d = 0.592). The total points were 32.83 at T2 and 29.48 points at T3. Four subjects improved more than nine points. All DHI results can be found in Supplementary Table 3.

#### Simulator Sickness

The SSQ showed a decrease immediately following each intervention session. The total points improved from 7.4 at the first session to 4.2 points at the last session. They mainly recorded “fullness of the head” and “sweating during the exercise.” Some noted general discomfort or moderate dizziness. During the evening hours of the same day, none of the participants experienced any symptom(s). The mean results of all SSQ measurements after each intervention session are presented in Supplementary Table 4.

#### Game Scores

The subjects' game high scores showed in the beginning continuous improvement in game performance. About half of the course of the intervention period there was a leveling off in performance increments. The game scores can be found Supplementary Figures 1A–H.

## Discussion

### Vestibular Compensation

The goal of this study was to evaluate the effects of a purpose-designed Exergaming rehabilitation program, including challenging conditions such as combined visual observation, head turns and unsupported stepping movements, on the vestibular compensation in subjects with cPVH. Based on the combined group and individual patient level assessment using an A-B research design there was clinically relevant improvement in some of the chronic subjects following the Exergame intervention phase. This might indicate that processes underlying central vestibular compensation were activated following the exergaming therapy in subjects with cPVH as opposed to non-observable change in the control phase. We found significantly smaller cumulative overt saccades amplitudes in more than half of the subjects. However, there was no change in VOR gain. A similar result was recently reported by Millar et al. (57). In their study, a progressive vestibular rehabilitation program was conducted with subjects after unilateral vestibular deafferentation surgery. Their subjects clinically and statistically improved despite absent change in VOR gain (57). The interventions compared to this study were different. The vestibular physical therapy used in the study by Millar et al. was five times longer (total of 945 min over five weeks) than the exergaming performed in this study (total of 176 min over four weeks).

The COSA in this study positively correlated with DVA. Thus, smaller COSA mean better visual acuity.

After the intervention, the correlation between COSA and VOR gains was no longer significant, suggesting that the patients were able to compensate their VOR deficit with covert saccades during the head impulses, which are not accounted for in the COSA measure.

#### Gait Function

The subjects' gait function showed significant improvements. The FGA improved significantly and five subjects improved their FGA scores by at least nine points, which lies well above the minimal detectable change value for this measure (49). This makes the chance of a measurement error less likely as possible alternative explanation. When the training principle of specificity applies, the improved FGA task “gait with horizontal head turns” might be caused by the training content of provoked head turns while exergaming. The exergaming led to a safer locomotion. It is important to stress that the individuals partaking in our program were all chronic subjects from whom further treatment effects were expected being more difficult to achieve (22) based on their chronicity status. Seeing an improvement in half of the chronic patients in our sample is encouraging for the relevance of exergaming as a novel intervention and important on an individual subjects' daily living level. Finally, subjects' game scores showed continuous improvement in game performance meaning they could play the Exergames better and faster over the intervention course.

### Study Limitations

The approach for the data analysis we used may be seen as a limitation of our study. The combination of group- and individual-level data analysis has intuitive appeal allowing individual-level associations through the mechanisms of bias control (AB design), the separation of contextual within and between-group effects, and the repeated checking of the treatment underlying model (58). However, such an approach theoretically necessitates knowledge about previous rehabilitation programs which each individual experienced as these may effect on the observable treatment effects of individuals (59). Gathering such detailed information for our participants was beyond the means of our study. A further limitation of the present study is the rather small sample size of 12 subjects that led to a weak statement regarding the population level, thus, limiting the generalizability of our treatment. However, in the current stage of intervention development we were mainly interested in individual patient responses to the intervention, since the expectation for these individuals to improve were rather low based on the chronicity of their symptoms and the years of previous therapy these individuals had undergone.

## Conclusion

The results of the present study demonstrate that exergaming with promoted head turns facilitates vestibular compensation in some subjects with cPVH. This is the first study that shows an improvement in cumulative overt saccade amplitude after exergaming in several chronic vestibular subjects. The findings of this study warrant further research in which more complex research designs in larger samples should be employed.

## Data Availability Statement

All datasets generated for this study are included in the article/Supplementary Material.

## Ethics Statement

The studies involving human participants were reviewed and approved by Ethics committee of the Canton of Zurich under BASEC 2018-00337. Trial registration was done at ClinicalTrials.gov Identifier; NCT03536533. The patients/participants provided their written informed consent to participate in this study.

## Author Contributions

JS developed the research question. The concept and design part were established by FB and JS. FB executed the intervention. FB, JS, and KW did the analysis of the results. KW, DS, and JS did the interpretation of the results. JS and FB produced an early version of the manuscript. EB, KW, and DS critically revised the manuscript to bring it to its current version. All authors have read and approved the final manuscript.

## Conflict of Interest

EB was a co-founder of Dividat; the spin-off company that developed the exergame platform used in this study, and is associated to the company as an external advisor. No revenue was paid (or promised to be paid) directly to EB or his institution over the 36 months prior to submission of the work. KW acts as an unpaid consultant and has received funding for travel from Otometrics. The remaining authors declare that the research was conducted in the absence of any commercial or financial relationships that could be construed as a potential conflict of interest.
