# Supplementary material for: Exergaming With Integrated Head Turn Tasks Improves Compensatory Saccade Pattern in Some Patients With Chronic Peripheral Unilateral Vestibular Hypofunction
Source: Front Neurol. 2020 Jun 30;11:601. doi: 10.3389/fneur.2020.00601 (PMC7344241; doi:10.3389/fneur.2020.00601)

Supplementary Material

Supplementary S1. The results of FGA all tasks and total score

	Baseline Mean	Post intervention Mean	*p*	Z
Gait on level surface; (SD)<br>Range	2.22 (0.67)<br>1/3	2.83 (0.39)<br>2/3	0.029	-3.08
Change in gait speed; (SD)<br>Range	2.33 (0.65)<br>1/3	2.83 (0.39)<br>2/3	0.030	-2.12
Gait with horizontal head turns, (SD)<br>Range	1.42 (0.67)<br>0/2	2.33 (0.49)<br>2/3	<0.001*	-2.12
Gait with vertical head turns; (SD)<br>Range	1.50 (0.67)<br>0/2	2.67 (0.50)<br>2/3	0.041	-2.64
Gait with pivot turn; (SD)<br>Range	2.25 (0.75)<br>1/3	2.67 (0.65)<br>1/3	0.065	-2.91
Step over obstacle; (SD)<br>Range	2.17 (0.94)<br>0/3	2.75 (0.45)<br>2/3	0.021	-1.89
Gait with narrow base of support;(SD)<br>Range	1.92 (1.08)<br>0/3	2.50 (0.52)<br>2/3	0.071	-2.33
Gait with eyes closed; (SD)<br>Range	1.17 (0.72)<br>0/2	2.08 (0.67)<br>1/3	<0.001*	-1.84
Ambulating backward; (SD)<br>Range	2.00 (0.74)<br>1/3	2.83 (0.39)<br>2/3	<0.001*	-3.05
Steps; (SD)<br>Range	2.42 (0.52)<br>2/3	2.67 (0.49)<br>2/3	0.084	-2.89
Total; (SD)<br>Range	19.58 (5.55)<br>8/26	26.17 (2.98)<br>18/29	<0.001*	-1.73

SD = standard deviation; Range = min/max; Z, p= Wilcoxen Signed Rank Test; *Bonferroni correction $p < 0.008$

Supplementary S2: The results of ETGUG all tasks and total score

	Baseline<br>Mean	Post intervention<br>Mean	*p*	Z
Sit-to-stand; sec (SD)<br>Range	1.95 (1.44)<br>0.95/6.40	1.32 (0.7)<br>0.20/2.50	0.59	-2.67
Gait initiation; sec (SD)<br>Range	1.54 (1.31)<br>0.65/5.40	1.17 (1.1)<br>0.50/4.20	0.55	-1.22
Walk 1; sec (SD)<br>Range	4.23 (1.41)<br>2.60/7.30	3.50 (1.18)<br>1.30/4.50	0.20	-0.77
Turn around; sec (SD)<br>Range	3.73 (1.37)<br>1.90/7.60	3.58 (1.18)<br>2.20/5.40	0.85	-1.57
Walk 2; sec (SD)<br>Range	4.80 (1.38)<br>3.10/7.80	3.49 (0.95)<br>1.80/5.50	0.03	-0.16
Slow down, stop, turnaround,<br>and sit down; sec (SD)<br>Range	3.65 (1.31)<br>2.0/6.60	2.48 (1.04)<br>1.80/4.30	<0.001*	-2.24
Total time; sec (SD)<br>Range, min/max	19.95 (5.14)<br>12.8 /31.00	15.61 (4.59)<br>7.30/22.20	<0.001*	-2.67

SD = standard deviation; Range = min/max; Z, p= Wilcoxen Signed Rank Test; *Bonferroni correction $p < 0.005$

Supplementary S3: DHI results

	Baseline<br>Mean	Post intervention<br>Mean	*p*	Z
Total points (SD)<br>Range	32.83 (23.76)<br>6/74	29.48 (26.95)<br>0/84	0.17	-1.38

SD = standard deviation; sec = seconds; *p* = Wilcoxen Signed Rank Test; * = significant exact difference (one-tailed) (p<0.05); Range = min/max

Supplementary S4: Mean Scores of the Simulator Sickness Questionnaire after each exercise session

	Exercise Sessions							
	1	2	3	4	5	6	7	8
Mean (SD)	7.4 (5.7)	5.2 (4.9)	6.0 (4.2)	6.1 (5.4)	4.8 (5.2)	3.0 (3.9)	4.8 (6.3)	4.2 (3.6)
Range	1/18	0/15	0/14	0/16	0/15	0/13	0/11	0/13

Supplementary Figure S1a-h Game score of the exergames **(units reflect points scored in the exergame)**

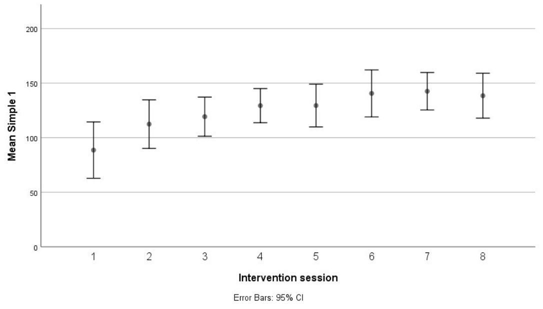

S1a Game Simple 1

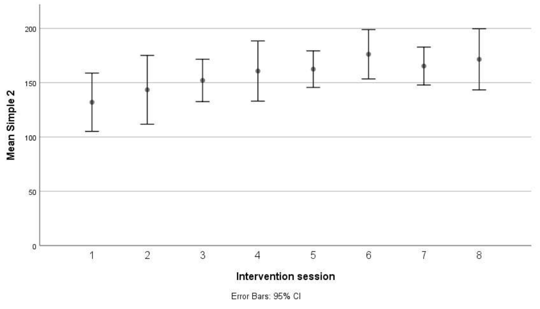

S1b Game Simple 2

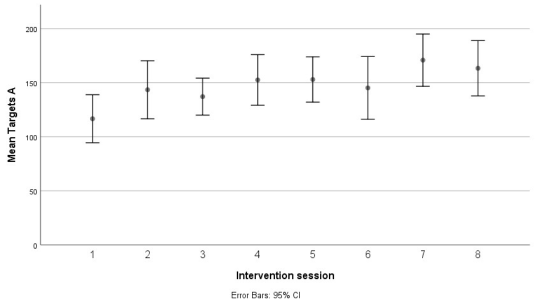

S1c Game Targets A

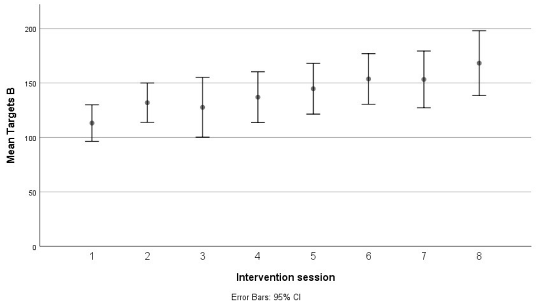

S1d Game Targets B

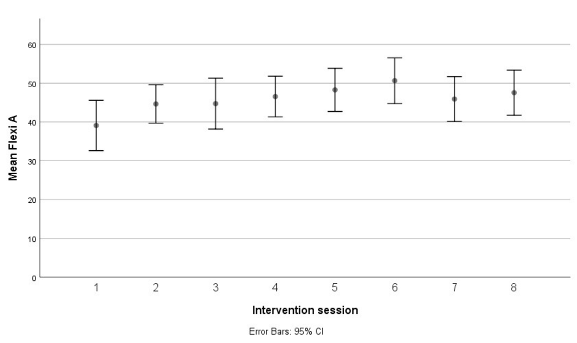

S1e Game Flexi A

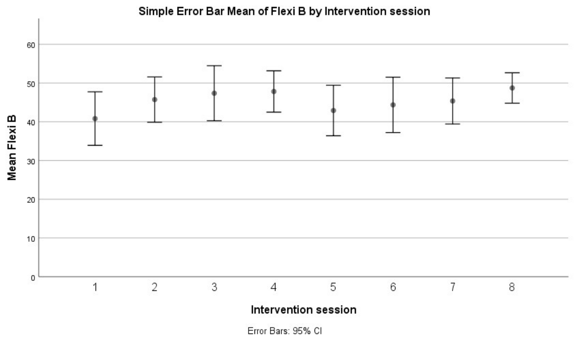

S1f Game Flexi B

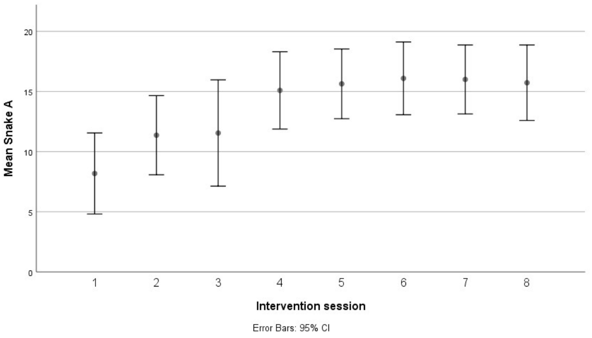	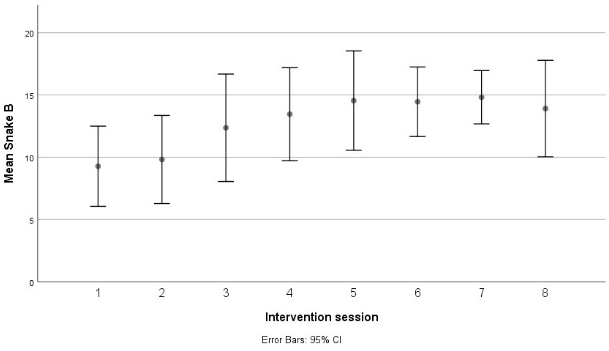
S1g Game Snake A	S1g Game Snake A

Figure S2 a-d

Figure 2a The game Simple trains focussed attention – the ability to concentrate on certain stimuli and react as quick as possible to them

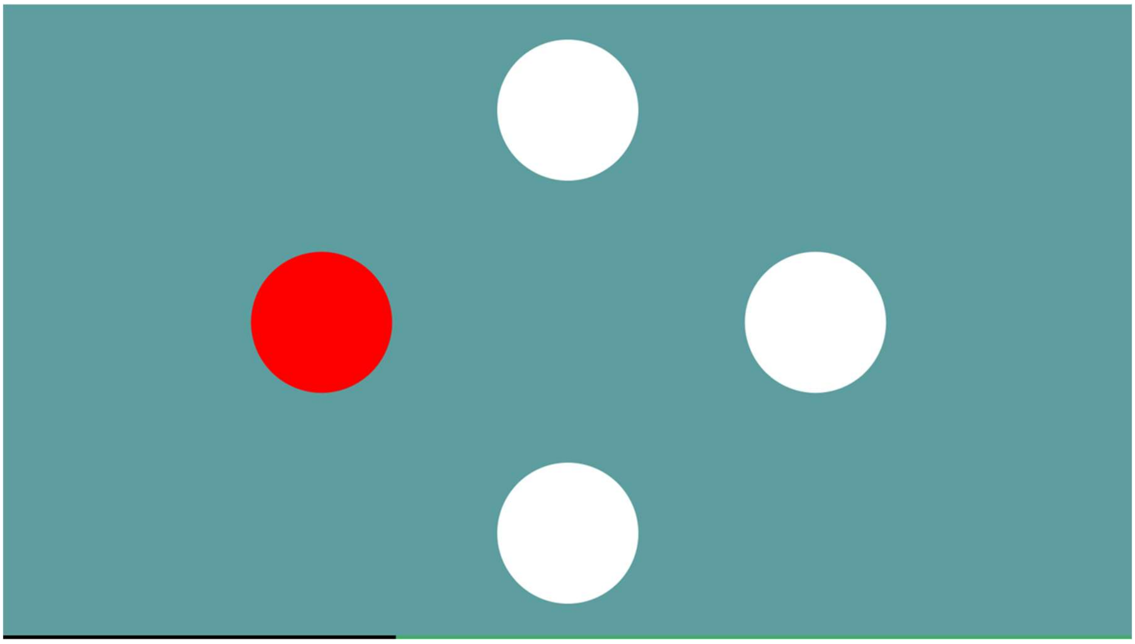

Figure 2b The game Targets which helps training reaction time (speed & accuracy)

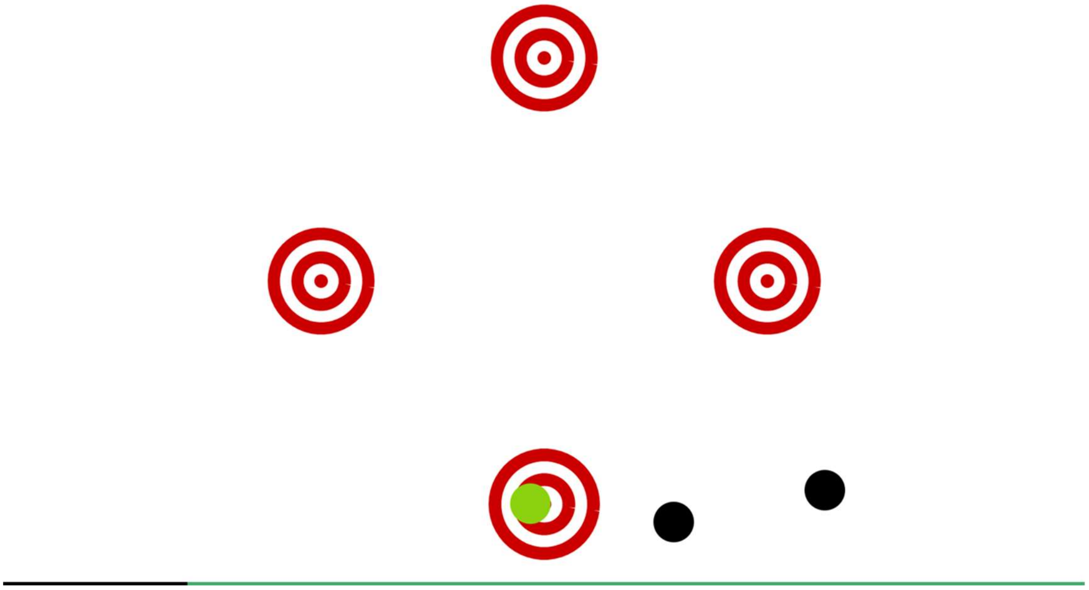

Figure 2c Flexi supports training of shifting attention

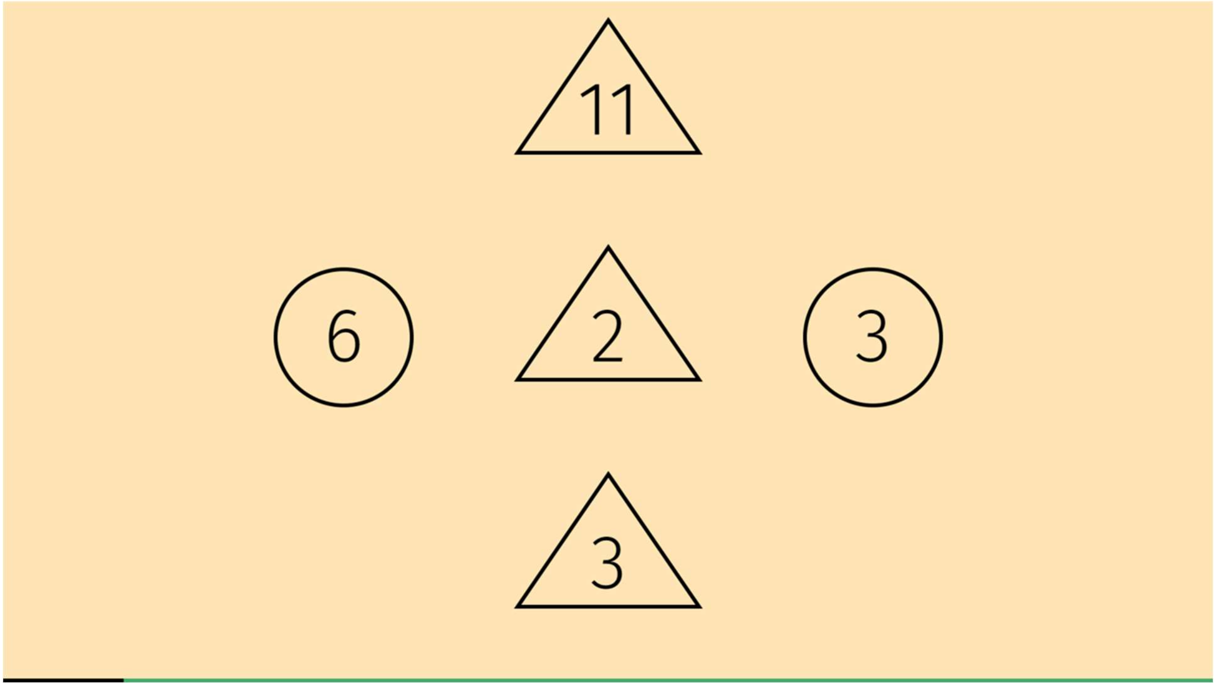

Figure 2d The Snake game which supports training of spatial orientation in a 2D virtual environment

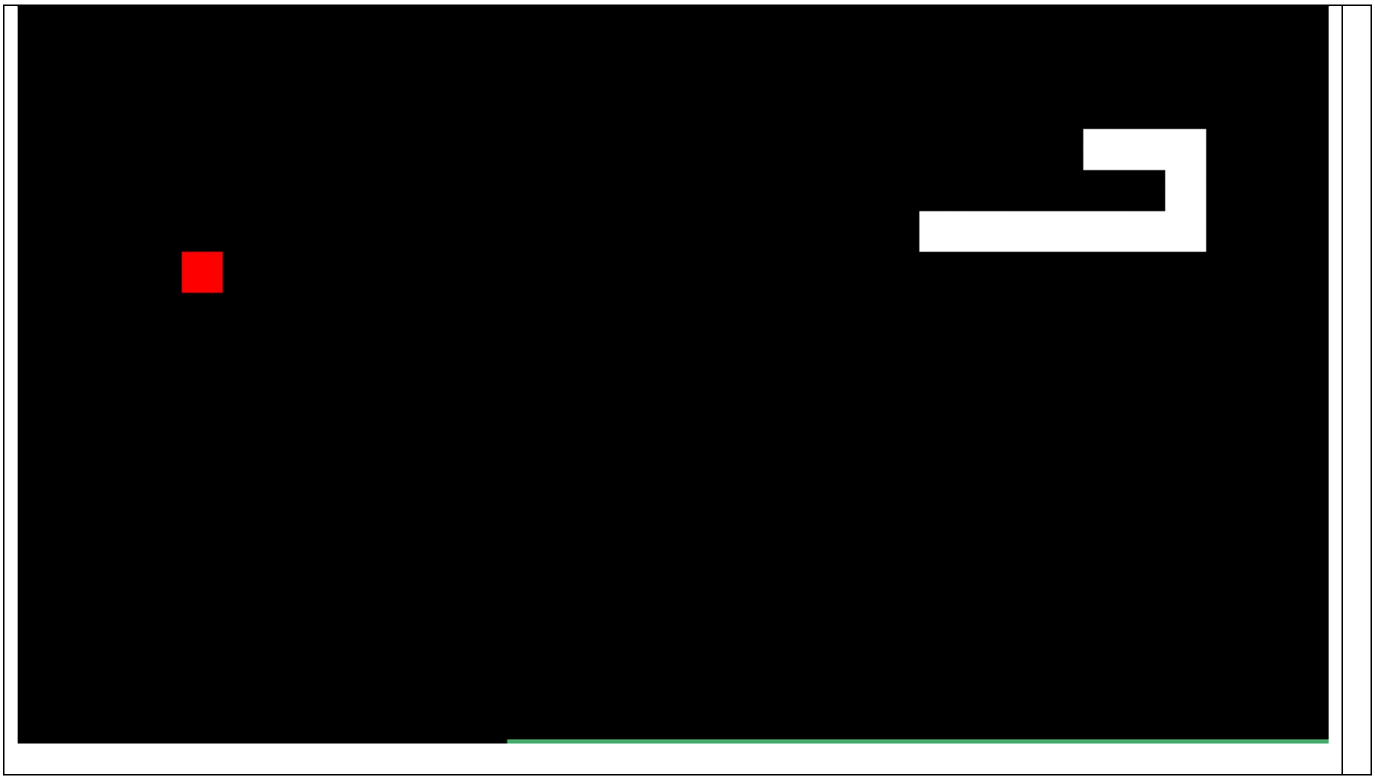

Supplement: Supplementary file 2 [file Data_Sheet_1.pdf]
